# The Atrial Fibrillation Registry (The FLOW‐AF Registry): Insights From the United Arab Emirates—Patient Characteristics, Treatment, and One‐Year Outcomes

**DOI:** 10.1111/jce.16598

**Published:** 2025-02-10

**Authors:** Moutaz El Kadri, Khalid AlMuti, Amrish Agrawal, Nooshin Bazargani, Mohamed Fathy Soliman Gamaleldin, Haytham Mohamed Ahmed, Omneya Hassanain, Natasha Khalife, Ghazi Radaideh, Mohamed Magdy, Wael A. Almahmeed

**Affiliations:** ^1^ Sheikh Khalifa Medical City Abu Dhabi United Arab Emirates; ^2^ Cleveland Clinic Abu Dhabi Abu Dhabi United Arab Emirates; ^3^ Fujairah Hospital, EHS Fujairah United Arab Emirates; ^4^ Dubai Hospital Dubai United Arab Emirates; ^5^ Pfizer Gulf Dubai United Arab Emirates; ^6^ IQVIA, Real‐World Evidence Dubai United Arab Emirates; ^7^ Rashid Hospital Dubai United Arab Emirates; ^8^ Al Qassimi Hospital Sharjah United Arab Emirates

**Keywords:** clinical outcomes, healthcare resource utilization, non‐valvular atrial fibrillation, patient characteristics, treatment patterns, UAE

## Abstract

**Background:**

The epidemiological landscape and treatment efficacy of atrial fibrillation (AF) in the Middle East, notably in the United Arab Emirates (UAE), remain under‐explored, presenting a distinct demographic and clinical pattern compared to Western populations. The FLOW‐AF Registry aimed to bridge this knowledge gap by examining the characteristics, treatment patterns, clinical outcomes, and healthcare resource utilization (HCRU) of newly diagnosed non‐valvular atrial fibrillation (NVAF) patients in the UAE, contributing to the scant literature on NVAF management in the region.

**Methods:**

This multicenter, prospective observational study enrolled patients diagnosed with NVAF across six sites in the UAE. Data were collected on demographics, medical history, treatment decisions, clinical outcomes, and HCRU over a 12‐month observation period.

**Results:**

A total of 198 patients were enrolled, with a mean age of 63.44 years. Mean CHA₂DS₂‐VASc and HAS‐BLED scores at baseline were 2.95 and 1.76, respectively. Most patients (55.77%) were prescribed non‐vitamin K antagonist oral anticoagulants (NOACs). One‐year incidence rates for major clinical events were 6.7% for all‐cause mortality, 2.8% for bleeding complications, and 0.6% for myocardial infarction. No strokes were reported during the study period.

**Conclusion:**

The FLOW‐AF Registry provides valuable insights into NVAF management in the UAE, demonstrating a distinct patient profile and a preference for NOACs. The NVAF cohort in the UAE was characterized by a younger demographic with lower risk scores and lower rate of clinical events relative to global counterparts. The findings underscore the evolving approach to AF management in the UAE, suggestive of a shift towards NOAC use but also highlights the need for ongoing research to fully understand long‐term outcomes and validate current treatment paradigms in the UAE.

## 1 Introduction

Arial Fibrillation (AF), the most prevalent sustained cardiac arrhythmia, affects approximately 37.6 million individuals worldwide, or 0.51% of the global population [1]. Characterized by uncoordinated atrial activation, AF significantly elevates the risk of stroke, transient ischemic attack (TIA), myocardial infarction (MI), and heart failure (HF) [2], and is responsible for nearly 15% of all strokes. Strokes associated with AF tend to be more severe, leading to heightened morbidity and mortality [3]. Nonvalvular atrial fibrillation (NVAF), which excludes cases with mechanical prosthetic heart valves or significant mitral stenosis, constitutes 77%–91% of all AF cases, underscoring its clinical importance [4, 5].

The incidence of AF is surging globally, paralleling increases in life expectancy [6]. AF prevalence shows marked age dependency, ranging from 0.1%–0.2% in individuals under 49 to 13.5%–17.8% in those aged 80 years and older [7]. From 1990 to 2019, the incidence and prevalence of AF worldwide more than doubled, from 2.1 to 4.7 million, and from 2.8 to 59.7 million, respectively, reflecting a growing public health challenge [8]. Projections suggest a more than 60% increase in AF burden by 2050, especially in regions with a middle socio‐demographic index, emphasizing the need for focused prevention and treatment strategies [6].

In the Middle East, AF incidence is rising, likely due to rapid economic growth, shifts towards Western diets, and increased metabolic and cardiovascular disease rates. Notably, AF patients in the Middle East present younger and with more comorbid conditions than their Western counterparts [6, 9]. A retrospective study using the Dubai Real World Claims Database (DRWD) from 2014 to 2017 reported a mean patient age of 59 years [10], highlighting the need for region‐specific research to guide clinical and policy decisions effectively.

The economic impact of AF is substantial. In the United States, annual AF‐related expenditures approach 26 billion USD, with direct AF treatment costs around 6 billion USD [11]. European annual expenses vary from 660 million to 3.3 billion EUR, primarily due to hospitalizations and stroke complications [12]. A 2013 study across three hospitals in Saudi Arabia (KSA) and the United Arab Emirates (UAE) found per‐person annual costs attributable to AF of 1151 USD in the UAE and 3001 USD in KSA, with significant resource utilization and cost discrepancies between the two countries [13]. These findings underscore the necessity for country‐specific data to inform healthcare policies effectively.

Stroke prevention, a critical component of AF management, has traditionally relied on oral anticoagulant (OAC) treatments like Vitamin K antagonists (VKAs). However, VKAs require frequent monitoring due to their narrow therapeutic range [14]. The advent of non‐vitamin K antagonist oral anticoagulants (NOACs) has offered a more convenient stroke prevention alternative, validated through randomized controlled trials demonstrating efficacy and safety [15, 16, 17]. Globally, the use of NOACs has significantly risen from 2010 to 2018, with prevalent and incident user rates increasing markedly [18]. Despite high NOAC prescription rates in Europe [19, 20], data on NOAC use in the Middle East (ME) and Africa remains limited. A shift towards NOACs in the DRWD from 2015 to 2019 suggests an increasing adoption over time [21].

The limited data on NVAF in the ME, including the UAE, necessitates comprehensive studies on current prescribing practices and resource utilization. The FLOW‐AF registry was established with the aim to evaluate the characteristics, treatment patterns, clinical outcomes, and healthcare resource utilization (HCRU) of newly diagnosed NVAF patients in Egypt, Lebanon, KSA, and UAE. This study seeks to provide contemporary data in these countries, contributing to the evolving field of NVAF management by offering insights into current practices and outcomes. In doing so, it aims to address the gap in region‐specific NVAF data, thereby informing clinical management and healthcare policy formulation in a region where comprehensive, up‐to‐date information remains sparse. This manuscript details the UAE cohort findings.

## 2 Materials and Methods

### 2.1 Study Design

The FLOW‐AF registry was a multicenter, prospective observational study that enrolled patients diagnosed with NVAF from 6 geographically representative sites across the UAE. This study was designed to capture real‐world data on NVAF management, patient outcomes, and HCRU. The study protocol and its comprehensive methodology have been detailed in a previous publication [22].

A 12‐month recruitment period was originally planned, however the recruitment timeline extended to approximately 28 months (March 2019 to June 2021), primarily due to delays caused by the COVID‐19 pandemic. Following enrollment, patients were observed for 12 months, with the total study period spanning from January 2019 to July 2022.

### 2.2 Study Population

Eligible participants were adults (≥ 18 years) newly diagnosed with NVAF within the enrollment period or 90 days before enrollment, who initiated treatment for stroke/systemic embolism (SE) prevention. Exclusions applied to those with unlikely follow‐up, involvement in interventional trials, OACs prescribed for conditions other than AF, severe psychiatric disorders, AF attributable to reversible causes, mechanical heart valves, severe valve disease, and pregnant or breastfeeding women.

### 2.3 Data Collection

Patient data were prospectively collected from medical records using electronic Case Report Forms (eCRFs) hosted on a secure online platform. Hospital‐level healthcare costs were collected using structured questionnaires, detailing costs by unit. Data were collected at baseline (enrollment), 6 months (± 2 months), and 12 months (± 2 months) post‐enrollment. Baseline data included patient demographics, NVAF‐disease specific information, medical history (comorbidities, CHA₂DS₂‐VASc and HAS‐BLED scores, lifestyle factors), treatment patterns, HCRU, and direct costs. Follow‐up data included clinical outcomes (stroke, TIA, SE, bleeding events, MI, mortality), HCRU, and associated costs.

### 2.4 Statistical Analysis

The target sample size of 227 patients was determined based on an expected frequency of 0.5 for any outcome, the estimated AF population size in UAE, margin of error of 0.07, and 10% attrition rate. Descriptive statistics were utilized for demographics, clinical characteristics and treatment patterns. Antithrombotic treatment data were stratified by baseline CHA₂DS₂‐VASc and HAS‐BLED scores. Kaplan‐Meier estimates were used for time‐to‐event outcomes, and incidence rates were expressed as events per patient‐year. Economic analyses focused on HCRU and associated costs. HCRU‐related costs were considered only when complete cost data were available. The number of dispensed anti‐thrombotic medication packages was estimated from package size, drug strength, daily dosage, and treatment duration, with any missing values imputed using the mean of available data for that specific drug. All other data were analyzed as recorded, without imputation. Statistical analyses were conducted using R software (version 4.1.1).

## 3 Results

### 3.1 Baseline Demographics, Clinical Characteristics, and Risk Factors

Based on the eligibility criteria, 198 patients (93.4% of those screened) were successfully enrolled, across 6 sites in the UAE, while 14 patients were excluded due to not being newly diagnosed (*n* = 3), having mechanical heart valves or valve disease expected to require valve replacement (*n* = 1), and not using antithrombotic medication at baseline (*n* = 10). Of the enrolled patients, 55.1% (*n* = 109) were men and 66.7% (*n* = 132) were Caucasian (including Arab). Most of the enrolled patients (87.9%, *n* = 174) attended public hospitals, while 12.1% (*n* = 24) attended semi‐governmental hospitals (hospitals accepting private and public insurances). The mean (SD) age of patients was 63.4 years (14.5). The mean (SD) systolic‐, diastolic‐ blood pressure, and heart rate were 131.6 (19.75) mmHg, 75.5 (12.80) mmHg, and 86.6 (25.00) beats/min, respectively. The majority of patients were either obese (*n* = 81, 43.78%) or overweight (*n* = 57, 30.8%), with a mean (SD) BMI of 30.0 kg/m^2^ (6.62). At baseline, most patients were reported as non‐smokers (*n* = 115, 67.7%), and reported to not consume alcohol (*n* = 159, 99.4%) (Table 1).

**Table 1:** Demographic characteristics and vital signs of patients at baseline.

Variables	Parameters	
Number of patients enrolled	*N*	198
Gender		
	Male	109 (55.05%)
	Female	89 (44.95%)
Race		
	Caucasian (including Arab)	132 (66.67%)
	Other caucasian (non‐Arab)	3 (1.52%)
	Asian	59 (29.80%)
	African (Black african)	1 (0.51%)
	Hispanic/Latino	1 (0.51%)
	Unknown	0 (0.00%)
	Other, specify	2 (1.01%)
Type of hospital		
	Public	174 (87.88%)
	Semi‐governmental	24 (12.12%)
Age at baseline (years)		
	Mean (SD)	63.44 (14.48)
	Median [Q1–Q3]	64.00 [55.25, 75.00]
	Min–Max	26.00–96.00
BMI (kg/m^2^)		
	Mean (SD)	30.03 (6.62)
	Median [Q1–Q3]	29.14 [24.65, 33.31]
	Min–Max	17.78–53.33
	Missing	13 (6.57%)
BMI (categorical)		
	Underweight (BMI < 18.5)	1 (0.54%)
	Normal weight (18.5 ≤ BMI < 25.0)	46 (24.86%)
	Overweight (25.0 ≤ BMI < 30.0)	57 (30.81%)
	Obese (BMI ≥ 30.0)	81 (43.78%)
	Missing	13 (6.57%)
Systolic blood pressure (mmHg)		
	Mean (SD)	131.61 (19.75)
	Median [Q1–Q3]	131.00 [117.00, 144.00]
	Min–Max	95.00–194.00
	Missing	1 (0.51%)
Diastolic blood pressure (mmHg)		
	Mean (SD)	75.49 (12.80)
	Median [Q1–Q3]	74.00 [66.00, 83.00]
	Min–Max	48.00–118.00
	Missing	1 (0.51%)
Heart rate (beats/min)		
	Mean (SD)	86.62 (25.00)
	Median [Q1–Q3]	81.00 [70.00, 97.00]
	Min–Max	38.00–156.00
	Missing	1 (0.51%)
Smoking habits defined at baseline		
	Current smoker	26 (13.13%)
	Former smoker	13 (6.57%)
	Non‐smoker	157 (79.29%)
	Passive smoking	2 (1.01%)
Alcohol consumption	No	193 (97.47%)
	Yes	5 (2.53%)

At baseline, the overall mean (SD) time since NVAF diagnosis was 12.8 (20.9) days. Electrocardiogram (ECG) was the most common method used for NVAF diagnosis (*n* = 177, 82.3%), followed by implantable devices (6.05%, *n* = 13). Holter monitor was used in 8 patients (3.7%) and 17 patients (7.9%) used other methods. The majority of patients did not have any family history (among first degree relatives) of NVAF (*n* = 191, 96.0%) nor stroke (*n* = 193, 98.0%). None of the patients had a family history of venous thromboembolism (Table 2).

**Table 2:** Atrial fibrillation characteristics at baseline.

Variables	Parameter	
Number of patients enrolled	*N*	198
Time (days) since NVAF diagnosis	*N*	198
	Mean (SD)	12.80 (20.84)
	Median [Q1–Q3]	4.00 [2.00, 11.75]
	Min–Max	1.00–90.00
Method of NVAF diagnosis	*N*	215
	Electrocardiogram	177 (82.33%)
	Implantable devices	13 (6.05%)
	Holter monitor	8 (3.72%)
	Other^a^	17 (7.91%)
Family history of related diseases among first degree relatives		
NVAF	*N*	197
	No	191 (96.95%)
	Yes	6 (3.05%)
	Missing	1 (0.51%)
Stroke	*N*	197
	No	193 (97.97%)
	Yes	4 (2.03%)
	Missing	1 (0.51%)
Venous thromboembolism	*N*	197
	No	197 (100.00%)
	Missing	1 (0.51%)

*Note:* Some patients had several methods for NVAF diagnosis.

a Other methods include discharge summary from patient admission, transesophageal echocardiogram, echocardiogram, physician's note, stress test, and telemetry.

The most common comorbidities were hypertension (70.2%, *n* = 139), diabetes mellitus (47.0%, *n* = 93), hypercholesterolemia (45.0%, *n* = 89), coronary artery disease (29.8%, *n* = 59), congestive HF/left ventricular dysfunction (25.8%, *n* = 51), coronary surgery/stenting (22.7%, *n* = 45), and MI (13.6%, *n* = 27). There were 15 (7.6%) patients with a history of stroke/TIA at baseline (Supporting Information S1: Table S1).

Most patients had a high CHA₂DS₂‐VASc stroke risk score of ≥ 2 (*n* = 155, 78.3%); 29 patients (14.7%) patients had an intermediate stroke risk score of 1, and 14 patients (7.1%) patients had a low stroke risk score of 0. The majority of patients fell within the low HAS‐BLED risk score category of < 3 (*n* = 150, 76.1%); 47 (23.9%) patients fell within the high HAS‐BLED risk score category of ≥ 3. Overall, the mean (SD) CHA₂DS₂‐VASc and HAS‐BLED risk scores were 3.0 (1.7) and 1.76 (1.2), respectively. Th most common modifiable risk factor for bleeding identified in the study was hypertension (*n* = 139, 70.2%). Anemia was the most common potentially modifiable risk factor (*n* = 26, 13.1%). Among non‐modifiable risk factors, age greater than 65 years was the most frequently observed (*n* = 92, 46.5%) (Supporting Information S1: Table S2).

### 3.2 Patterns of Care of Newly Diagnosed Patients With NVAF

At baseline, all 198 patients received antithrombotic treatments. In total, there were 260 antithrombotic treatments prescribed, and the most frequently prescribed were NOACs (73.2%, *n* = 145) and antiplatelet therapies (42.4%, *n* = 84). Of the 84 prescribed antiplatelet therapies, 49 (58.33%) were prescribed alone and 35 (41.67) were prescribed with NOAC/VAC.

VKAs accounted for only 8.6% (*n* = 17) of antithrombotic treatments prescribed. Among the NOACs, apixaban was most prescribed (56.6%, *n* = 82), followed by rivaroxaban (33.1%, *n* = 48), and dabigatran (10.3%, *n* = 15). Of the antiplatelet therapies, aspirin accounted for 50% (*n* = 42) of the treatments, followed by clopidogrel (46.4%, *n* = 39), and ticagrelor (2.4%, *n* = 2). Warfarin was the only prescribed VKA (Supporting Information S1: Table S3).

Apixaban was used as 2.5 mg twice a day (BID) in 38 patients (46.3%), 5 mg BID in 36 patients (43.9%), and 5 mg once a day (QD) in 8 patients (9.8%), for a mean (SD) treatment duration of 228.00 (80.8) days. Rivaroxaban was used QD in all patients (*n* = 48) at a dose of 20 mg in 38 patients (79.2%) and 15 mg in 10 patients (20.8%), for a mean (SD) treatment duration of 189.1 (141.2) days (Supporting Information S1: Table S3).

**Figure 1:**
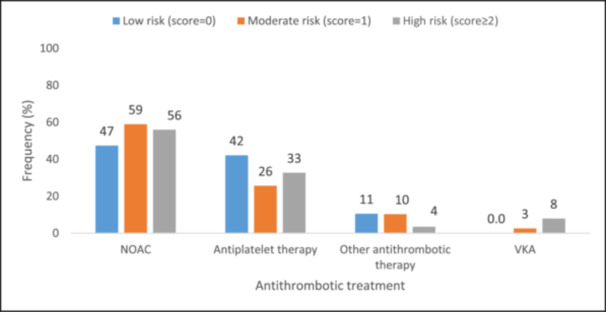
Antithrombotic treatment at baseline by CHA₂DS₂‐VASc score. NOAC, non‐vitamin K antagonist oral anticoagulants; VKA, vitamin K antagonist.

**Figure 2:**
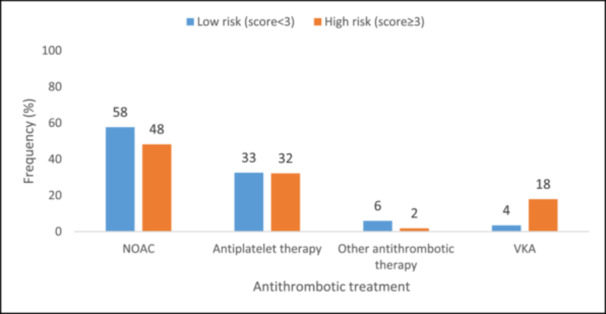
Antithrombotic treatment at baseline by HAS‐BLED score. NOAC, non‐vitamin K antagonist oral anticoagulants; VKA, vitamin K antagonist.

NOAC was the most frequently prescribed medication (≥ 47%) across all CHA₂DS₂‐VASc risk categories at baseline, followed by antiplatelet therapy (> 25%). VKAs were not prescribed to patients with low CHA₂DS₂‐VASc stroke risk score (score 0) (Figure 1). NOACs were also the most commonly prescribed medications (≥ 48%) across HAS‐BLED score groups (score < 3 and score ≥ 3) at baseline, followed by antiplatelet therapy (≥ 32%) (Figure 2).

NOACs were the most common antithrombotic treatments used at first‐line (56.0%, *n* = 145) and second‐line (93.0%, *n* = 13). Among NOACs, apixaban and rivaroxaban were the most frequently prescribed across both lines of treatment. Apixaban was used in 56.7% (*n* = 82) of the first‐line treatments and 84.6% (*n* = 11) of the second‐line, while rivaroxaban was used in 33.1% (*n* = 48) of first‐line and 7.7% (*n* = 1) of second‐line therapies. Approximately 7.0% (*n* = 17) of first‐line treatments and none of second‐line treatments were VKAs (Warfarin). Anti‐platelet therapy constituted 32.0% of first‐line treatments and 7% of second‐line treatments. Aspirin was the most common anti‐platelet therapy used in first‐line (50%, *n* = 42) and the only second‐line anti‐platelet therapy used in only one patient. Clopidogrel was the second most frequently prescribed anti‐platelet agent, representing 46.4% (*n* = 39) of the first‐line therapies (Table 3).

**Table 3:** First‐line, second‐line, and subsequent lines of antithrombotic treatment for stroke prevention in NVAF patients^a^.

Variables	Line of treatment	
	First line	Second line
Number of patients receiving antithrombotic treatment	*N* = 198	*N* = 14
Number of drugs at baseline	*N* = 260	*N* = 14
Oral anticoagulant: NOAC, *N* (%)	145 (55.77%)	13 (92.86%)
Apixaban	82 (56.55%)	11 (84.62%)
Rivaroxaban	48 (33.10%)	1 (7.69%)
Dabigatran	15 (10.34%)	1 (7.69%)
Edoxaban	0 (0.00%)	—
Oral anticoagulant: VKA, *N* (%)	17 (6.54%)	0 (0.00%)
Warfarin	17 (100.00%)	0 (0.00%)
Unknown	0 (0.00%)	—
Acenocoumarol	0 (0.00%)	—
Anti‐platelet therapy, *N* (%)	84 (32.31%)	1 (7.14%)
Clopidogrel	39 (46.43%)	0 (0.00%)
Aspirin	42 (50.00%)	1 (100.00%)
Ticagrelor	2 (2.38%)	—
Dipyridamole	1 (1.19%)	—
Other antithrombotic therapy, *N* (%)	13 (5.00%)	0 (0.00%)
Enoxaparin	13 (100%)	0

Abbreviations: NOAC, new oral anti‐coagulants; VKA, vitamin K antagonists.

a The “first line” referred to the initial antithrombotic therapy prescribed to newly diagnosed patients with NVAF at baseline. The “first line” encompassed the primary regimen initiated before any subsequent treatment changes (e.g., a switch or addition of therapy).

At baseline, the majority of patients (90.9%, *n* = 180) were prescribed at least one additional treatment in conjunction with their primary therapy. Lipid‐lowering drugs were the most common concomitant treatment, administered to 64.6% (*n* = 128) of patients, followed by beta‐blockers (60.1%, *n* = 119) and other anti‐hypertensive drugs (43.9%, *n* = 87). During the follow‐up period, 21.2% (*n* = 42) of patients received at least one concomitant treatment, of which the most common were beta‐blockers (41.0%) and other anti‐hypertensive drugs (36.0%) (Table 4).

**Table 4:** Concomitant treatments ongoing at baseline and during follow‐up periods.

	Ongoing at baseline	During the follow‐up period
Number of patients who received at least 1 concomitant treatment	180	42
Anti‐arrhythmics	27 (15.0%)	5 (11.9%)
Amiodarone	18 (66.7%)	3 (60.0%)
Flecainide	0	1 (20.0%)
Propafenone	4 (14.8%)	0
Other	5 (18.5%)	1 (20.0%)
Lipid‐lowering drugs	128 (71.1%)	7 (16.7%)
Beta‐blockers	119 (66.1%)	17 (40.5%)
Bisoprolol	102 (85.7%)	14 (82.4%)
Carvedilol	8 (6.7%)	0
Metoprolol	7 (5.9%)	0
Nebivolol	1 (0.8%)	0
Other	2 (1.7%)	4 (23.5%)
Calcium channel blockers	42 (23.3%)	3 (7.1%)
Verapamil	1 (2.4%)	0
Other	41 (97.6%)	3 (100.0%)
Cardiac glycosides	5 (2.8%)	1 (2.4%)
Digoxin	4 (80.0%)	1 (100.0%)
Other	1 (20.0%)	0
Other anti‐hypertensive drugs	87 (48.3%)	15 (35.7%)
Nonsteroidal anti‐inflammatory drugs	2 (1.1%)	1 (2.4%)
Oral antidiabetic drugs	56 (31.1%)	6 (14.3%)
Insulin	23 (12.8%)	2 (4.8%)
Anxiolytics	1 (0.6%)	1 (2.4%)
Antidepressants	2 (1.1%)	0
Synthetic thyroid hormones	8 (4.4%)	0
Hormone replacement therapy	1 (0.6%)	0
Bisphosphonates	1 (0.6%)	0
Antibiotics	1 (0.6%)	1 (2.4%)

### 3.3 Clinical Outcomes

Overall, 17 (9.6%) patients experienced at least one clinical outcome: bleeding (*n* = 5, 2.8%), MI (*n* = 1, 0.6%), and all‐cause mortality (*n* = 12, 6.7%). Among the patients who experienced bleeding events, one had a major bleed, three had clinically relevant non‐major bleeds, and one had a minor bleed. All were on NOACs without concurrent dual antiplatelet therapy. The major bleed occurred in a patient on rivaroxaban. Clinically relevant non‐major bleeds were seen in patients on apixaban, clopidogrel, and one patient on warfarin. Another warfarin‐treated patient had a minor bleed. It was not possible to compute median (95% CI) estimates for time to first event for the clinical outcomes due to the small number of events that occurred during the follow‐up period. No events of stroke, TIA, or SE were reported during the study period.

### 3.4 NVAF‐Related HCRU and Direct Costs Associated With the Management of NVAF Patients

Most patients had laboratory assessments (*n* = 188, 94.95%), imaging exams (*n* = 171, 86.36%) and outpatient visits (*n* = 114; 57.58%) during the study period. The mean (SD) number of laboratory assessments and imaging exams were 20.4 (23.0) tests per patient per year (PPPY), and 7.4 (5.8) exams PPPY, respectively. The mean number of outpatient visits was 5.1 (5.2) visits PPPY. The mean (SD) number of antithrombotic medications (dispensed packages) was 11.1 (5.7) PPPY. The unit cost for inpatient admissions was highest (USD 425.5) of all the healthcare resources used, followed by surgical/non‐surgical procedures (USD 372.4) and antithrombotic medications (USD 86.0). The highest total costs were related to antithrombotic medication use, with a total cost of USD 1209.4 PPPY. This was followed by the total cost of inpatient admissions (USD 559.7 PPPY) and surgical/non‐surgical procedures (electrical cardioversion, ablation, and other procedures) (USD 530.8 PPPY) (Table 5).

**Table 5:** Economic burden associated with the management of patients with NVAF over the follow‐up period.

Number of patients enrolled and starting the follow‐up with the treatment in question		*N* = 198
Laboratory assessments in total		
Patients that had laboratory assessment(s) per year	*N*	188
Laboratory assessments/patient/year	Mean (SD)	20.4 (23.0)
Unit cost of a laboratory assessment [USD]	*N*	6.9^a^
Total cost of laboratory assessments/patient/year [USD]	Mean (SD)	Too few events
Imaging Exams, in total		
Patients that had exam(s) per year	*N*	171
Number of exams/patient/year	Mean (SD)	7.4 (5.8)
Unit cost of an exam [USD]	*N*	38.7^a^
Total cost of exams/patient/year [USD]	Mean (SD)	76.6 (86.1)
Surgical/non‐surgical procedures, in total		
Patients that had procedure(s) per year	*N*	30
Number of procedures/patient/year	Mean (SD)	1.6 (1.0)
Unit cost of a procedure [USD]	*N*	372.4^a^
Total cost of procedures/patient/year [USD]	Mean (SD)	530.8 (502.5)
Inpatient admissions, in total		
Patients that had inpatient admission(s) per year	*N*	36
Number of inpatient admissions/patient/year	Mean (SD)	1.3 (0.6)
Unit cost of an inpatient admission [USD]	*N*	425.5^a^
Total cost of inpatient admissions/patient/year [USD]	Mean (SD)	559.7 (279.1)
Outpatient visits, in total		
Patients that had outpatient visit(s) per year	*N*	114
Number of outpatient visits/patient/year	Mean (SD)	5.1 (5.2)
Unit cost of an outpatient visit [USD]	*N*	32.6^a^
Total cost of outpatient visits/patient/year [USD]	Mean (SD)	171.2 (243.9)
Other events, in total		
Number of patients that had other event(s) per year	*N*	6
Number of other events/patient/year	Mean (SD)	1.2 (0.4)
Unit cost of other event [USD]	*N*	—
Total cost of other events/patient/year [USD]	Mean (SD)	Too few events
Antithrombotic medications, in total		
Patients that had medication(s) per year	*N*	188
Number of medications/patient/year	Mean (SD)	11.1 (5.7)
Unit cost of a medication [USD]	*N*	86.0^a^
Total cost of medications/patient/year [USD]	Mean (SD)	1209.4 (659.7)

*Note:* Only included patients with at least one follow‐up visit. With exception of antithrombotic medications, each section summarizes patients that had at least one recorded healthcare resource utilization.

a No unique unit cost.

## 4 Discussion

The FLOW‐AF Registry, a multi‐country, prospective observational study, was established to address the critical gap in real‐world data on NVAF in the Middle East. The registry collected comprehensive data on treatment patterns, clinical outcomes, and HCRU among newly diagnosed NVAF patients in Egypt, UAE, Lebanon, and KSA [22]. It is imperative to assess NVAF management within the Middle Eastern context given the geographical, clinical practice, and demographic differences compared to Western countries [23, 24, 25]. This manuscript presents the findings from the UAE cohort, offering an insight into NVAF characteristics and management in the country.

The mean age of patients diagnosed with AF was 63.44 years. This observation aligns with findings from studies across the Gulf and Middle East regions [10, 13, 21, 26, 27, 28]. In contrast, investigations from the United States [23], Europe [24], and Canada [25] have documented a higher mean age at diagnosis, generally about 10 years older than our results. This discrepancy merits further investigation to understand regional variations in AF onset.

The UAE cohort exhibited a high prevalence of hypertension (70%), diabetes mellitus (47%), hypercholesterolemia (45%), and coronary artery disease (30%). Notably, the burden of diabetes mellitus among this sample of NVAF patients in UAE (47%) is more than twice the burden reported in the West (e.g., around 20%) [29, 30]. Findings from this study as well as previous research collectively suggest that comorbidities (such as diabetes, hypertension, cardiovascular conditions, obesity) add to the clinical burden in the NVAF population across the ME region [10, 13, 21, 31]. This highlights the unique health characteristics of the region compared to Western countries, emphasizing the importance of tailored strategies for managing NVAF in the ME setting.

In this study, most patients (78%) exhibited a CHA₂DS₂‐VASc stroke risk score of 2 or higher, with an average score of 2.95, which is consistent with findings from other research conducted in UAE where 66% of patients reported score of 2 or higher, and the broader ME region with an average score of 2.4 [13, 21]. However, the score is lower than that reported in other regions such as Asia, Europe, and Latin America (overall mean [SD] score: 3.5 ± 1.7 with 87.8% patients having CHA₂DS₂‐VASc stroke risk score ≥ 2) [32]. The relatively lower mean score in this cohort may be attributed to the younger average age (63.44 years), coupled with the fact that most patients did not have prior history of stroke/TIA or congestive heart failure (CHF). Furthermore, the majority of the study participants (76%) fell within the low HAS‐BLED risk score category of < 3 (mean score: 1.76). Regional variation in the mean scores was reported with ME/African reporting a lower mean scores (1.6 ± 1.0), suggesting a lower risk compared to Eastern Europe and East Asia regions (mean scores of 2.0 ± 1.2 and 2.1 ± 1.2, respectively) [32, 33].

The study findings indicate a preference for OAC which constituted 63% of all antithrombotic treatments at baseline, with NOACs constituting 56% compared with VKA (7%). Also, antiplatelet therapy contributed to nearly one‐third (32%) of the overall antithrombotic treatments. Treatment preferences observed in this study follow a similar trend to those reported in other ME studies [10, 21]; however, findings from other regions (North America, Europe, Asia, Latin America, and Europe) have reported more frequent use of VKAs (≥ 32%) [34, 35, 36]. NOAC was most commonly prescribed across all the CHA₂DS₂‐VASc stroke risk score groups (≥ 47%) and HAS‐BLED score groups (≥ 48%) at baseline. Apixaban (≥ 57%) and rivaroxaban (≥ 8%) were the most frequently prescribed NOACs among the NVAF patients across first‐line as well as second‐line. The overall treatment pattern observed in this study was in line with the international AF management guidelines and recommendations [37, 38]. Of note, VKA was not prescribed to patients with low CHA₂DS₂‐VASc stroke risk score (score 0) in this study. This could be due to the fact that VKA has been associated with a negative net clinical benefit among patients with low‐risk status (CHA₂DS₂‐VASc stroke score = 0) [39].

A study conducted in the UAE between 2005 and 2012 reported that NOAC usage was notably low, with less than 10% of patients receiving these medications [13]. In stark contrast, findings from this study indicate a substantial shift, showing that NOACs accounted for 56% of treatments at baseline, underscoring a significant uptrend in NOAC adoption. This aligns with research based on data extracted from the Dubai Real‐World Claims Database [21], spanning 2015–2019, which suggests an increasing acceptance and utilization of NOACs among NVAF patients [21]. Such a paradigm shift is supported by the FLOW‐AF UAE cohort which suggests a progressive transformation in anticoagulation management practices within the region. Collectively, the findings of this study along with other research suggest an evolving landscape of NOAC use in the UAE, potentially driven by evolution in clinical guidelines, enhanced drug availability, and heightened awareness of NOAC efficacy and safety profiles.

In the present investigation, apixaban was used as 2.5 mg BID or 5 mg BID by the majority of patients, which are the two recommended doses for regular and special patient groups. Compliance with dosing recommendations for apixaban could not be determined, since the prescribed doses were not compared to patient characteristics (age, body weight, serum creatinine) to assess the suitability of each dose to the concerned patient. Likewise, rivaroxaban was used as 15 or 20 mg QD by all patients, but compliance with dosing recommendations was not assessed through matching the prescribed dose with each patient's creatinine clearance.

The rates of 1‐year all‐cause mortality (6.7%) and bleeding events (2.8%) in this study were lower than data reported in most of the other studies from ME, Asia, and rest of the world (5.2%–15.3% and 2.0%–5.8%, respectively) [37, 38, 39, 40, 41, 42]. Whereas the proportion of CV mortality (MI: 0.6%) was considerably lower in this study compared with the results from other studies from ME, Asia, and rest of the world (4.0%–7.0%). [37, 38, 39, 40, 41, 42]. Moreover, no events of stroke, TIA, or SE were reported during the study period. This may be explained by several factors, including effective management of patients as demonstrated by the high use of NOAC, younger patient population, and a relatively low mean baseline CHA₂DS₂‐VASc score. Due to the improved safety and lower mortality, it has been suggested that NOAC should be the preferred treatment option for patients with AF starting OAC for stroke prevention, even in healthcare settings where VKA treatment is of high quality [43].

Antithrombotic medications were the major contributors to per‐patient total yearly cost (USD 1209.4) of NVAF management, followed by inpatient admissions (USD 559.7) and surgical/non‐surgical procedures (USD 530.8). A prior database study reported a mean all‐cause healthcare cost of USD 9747 during a 6‐month follow‐up period among NVAF patients; however, it did not derive the results related to different healthcare resources such as overall cost of antithrombotic medications, cost of hospitalizations, surgical procedures [21]. A retrospective chart review study that assessed HCRU among AF patients in UAE reported a mean total annual medication costs of 508.3 per patient which is less than half the medication cost from the FLOW‐AF study probably due to the difference in prescribing trends [13]. Due to limited published data describing the HCRU/related costs for NVAF patients in the UAE and across the ME, it is challenging to provide a holistic overview of the economic burden associated with NVAF.

In this study, medication cost having a higher total annual cost compared to inpatient admissions is an unusual finding which may be attributed to various factors including the lack of detailed data on medication strengths and packaging sizes necessitated reliance on estimations, which could have led to inaccuracies in the reported medication costs. Additionally, the impact of the COVID‐19 pandemic on the frequency of hospital and clinic visits may have skewed HCRU cost estimates. These factors underscore the need for a cautious interpretation of the HCRU and cost findings of this study. Despite these constraints, the research provides crucial insights into HCRU and associated costs for NVAF patients in the UAE, laying the groundwork for further detailed investigations.

Due to its observational design, this study inherently encounters challenges including the potential for bias and confounding factors, as well as limitations in data availability. The study prospective design might have also influenced the management practices at participating sites due to awareness of the study's objectives. This awareness could affect the generalizability of the results to the broader NVAF management context in the UAE. Moreover, the 12‐month follow‐up period further limits the evaluation of long‐term treatment outcomes.

The COVID‐19 pandemic posed additional challenges, impacting participant recruitment and follow‐up which could have affected the representativeness of the study cohort and the completeness of the data collected. This necessitates careful consideration when interpreting the study's clinical and economic outcomes, particularly the cost data, due to potential under‐reporting of healthcare utilization and the need to use inferred data for drug specifics owing to the data limitations.

Additionally, the study did not capture specific clinical reasons for prescribing NOACs to patients with a CHA2DS2‐VASc score of zero or for the off‐label once‐daily dosing of apixaban. These observed practices likely reflect real‐world deviations from guideline recommendations, influenced by individualized patient considerations or local clinical preferences. This lack of detailed documentation on the rationale behind such prescribing decisions is a key limitation that should be acknowledged when interpreting the study findings.

## 5 Conclusion

The FLOW‐AF Registry sheds light on the management and outcomes of NVAF within the ME, including the UAE. Patients with NVAF in UAE demonstrate unique demographic and clinical characteristics, including younger average age, low baseline CHA₂DS₂‐VASc and HAS‐BLED risk scores, and a higher prevalence of comorbid conditions (e.g., diabetes and obesity), compared with the NVAF population in Western countries. The registry also suggests a preference for NOACs over traditional VKAs, aligning with a worldwide shift towards therapies that enhance safety, effectiveness, and patient convenience. These results highlight the necessity of tailoring treatment strategies to regional characteristics. Nonetheless, there is a clear need for additional research with extended follow‐up periods to comprehensively evaluate NVAF treatment patterns and long‐term outcomes in UAE.

## Ethics Statement

The study was conducted in accordance with the principles of the Declaration of Helsinki, local regulatory requirements, and the International Conference on Harmonization guidelines for Good Clinical Practice.

## Consent

All participants provided written informed consent.

## Conflicts of Interest

M.E.K. received honoraria from Alnaghi, Bayer, and Pfizer for giving educational lectures in conferences and funding of educational activities from Medtronic. K.A., A.A., N.B., G.R., M.M., and W.A.A. have no conflicts to disclose. N.K. and O.H. are employees of IQVIA, which conducted the study on behalf of the sponsor. M.F.S.G. and H.M.A.M. are employees of Pfizer Inc. the study sponsor.

## Supporting information

Supporting information.

## Data Availability

The data that support the findings of this study are available on request from the corresponding author. The data are not publicly available due to privacy or ethical restrictions. The data that support the findings of this study are not publicly available due to privacy or ethical restrictions.
