# Supplementary material for: The Atrial Fibrillation Registry (The FLOW‐AF Registry): Insights From the United Arab Emirates—Patient Characteristics, Treatment, and One‐Year Outcomes
Source: J Cardiovasc Electrophysiol. 2025 Feb 10;36(4):813–23. doi: 10.1111/jce.16598 (PMC11984346; doi:10.1111/jce.16598)
Supplement: Supplementary file 1 — Supporting information. [file JCE-36-813-s001.docx]

**Table S1:** **Medical history**

| **Comorbidities** | **Patients having the comorbidity n (%)** |
| --- | --- |
| Number of patients enrolled | 198 |
| Number of patients with no significant medical history | 16 |
| Hypertension | 139 (70.20%) |
| Hypercholesterolemia | 89 (44.95%) |
| Diabetes Mellitus | 93 (46.97%) |
| Coronary Arterial Disease | 59 (29.80%) |
| Congestive Heart Failure/ Left ventricular Dysfunction | 51 (25.76%) |
| Coronary surgery/ Stenting | 45 (22.73%) |
| Chronic kidney disease | 33 (16.67%) |
| Myocardial infarction | 27 (13.64%) |
| Anemia | 26 (13.13%) |
| Renal failure | 19 (9.60%) |
| Stroke/ TIA (Transient ischemic attack) | 15 (7.58%) |
| Dialysis-dependent kidney disease | 11 (5.56%) |
| Other Endocrine disorders | 10 (5.05%) |
| Malignancy | 9 (4.55%) |
| Peripheral Arterial disease | 8 (4.04%) |
| Asthma | 8 (4.04%) |
| Other Renal disease | 8 (4.04%) |
| Other Respiratory disease | 5 (2.53%) |
| Cerebrovascular Disease | 5 (2.53%) |
| Valvular Diseases | 5 (2.53%) |
| Obstructive Sleep Apnea | 4 (2.02%) |
| Chronic obstructive pulmonary disease (COPD) | 4 (2.02%) |
| Bleeding History | 4 (2.02%) |
| Autoimmune disease | 4 (2.02%) |
| Peripheral vascular disease/ stenting/surgery | 3 (1.52%) |
| Any genetic factors considered to have an increased bleeding risk | 3 (1.52%) |
| Other Cardiovascular disease | 3 (1.52%) |
| Aortic plaque | 2 (1.01%) |
| Venous Thromboembolism | 2 (1.01%) |
| Abnormal liver function | 6 (3.03%) |
| Carotid surgery/stenting | 1 (0.51%) |
| Cirrhotic liver disease | 1 (0.51%) |

**Table S2:** **Bleeding risk factors of patients at baseline**

| **Risk Factor** | **Patients with the risk factor**  **n (%)** |
| --- | --- |
| **Modifiable bleeding risk factors** |  |
| Hypertension (especially when systolic blood pressure is >160 mmHg) | 139 (70.20%) |
| Labile INR or time in therapeutic range <60% in patients on  vitamin K antagonists | 3 (1.52%) |
| Medication predisposing to bleeding, such as antiplatelet  drugs and non-steroidal anti-inflammatory drugs | 38 (19.19%) |
| Current alcohol consumption (number of drinks/ week);  Excess of alcohol consumption is defined as ≥8 drinks/week | 5 (2.53%) |
| **Potentially modifiable bleeding risk factors** |  |
| Anemia | 26 (13.13%) |
| Impaired renal function | 40 (20.20%) |
| Impaired liver function | 8 (4.04%) |
| Reduced platelet count or function | 3 (1.52%) |
| **Non-modifiable bleeding risk factors** |  |
| Age > 65 | 92 (46.46%) |
| History of major bleeding | 4 (2.02%) |
| Previous stroke | 15 (7.58%) |
| Dialysis-dependent kidney disease or renal transplant | 11 (5.56%) |
| Cirrhotic liver disease | 1 (0.51%) |
| Malignancy | 9 (4.55%) |
| Genetic factors | 3 (1.52%) |

**Table S3:** **Antithrombotic treatment for stroke prevention in NVAF patients at baseline**

| **Number of patients** |  |  | 198 |
| --- | --- | --- | --- |
| **Number of drugs** |  |  | 260 |
|  |  |  |  |
| **Anti-thrombotic therapy class** | | | |
| **Oral Anticoagulant: NOAC** |  | N | 145 (55.77 %) |
| **Apixaban** |  | N | 82 (56.55 %) |
|  |  |  |  |
|  | **Frequency** | N | 82 |
|  |  | QD | 8 (9.76%) |
|  |  | BID | 74 (90.24%) |
|  |  | TID | 0 (0.00%) |
|  |  | QID | 0 (0.00%) |
|  |  | PRN | 0 (0.00%) |
|  |  | Other | 0 (0.00%) |
|  |  | Missing | 0 (0.00%) |
|  |  |  |  |
|  | **Total daily dose** | N | 82 |
|  |  | Mean (SD) | 7.20 (2.50) |
|  |  | Median [Q1 - Q3] | 5.00 [5.00,10.00] |
|  |  | Min-Max | 5.00 - 10.00 |
|  |  | Missing | 0 (0.00 %) |
|  |  |  |  |
|  | **Dosing schedule (frequency-mg)** | N | 82 |
|  |  | QD-5 | 8 (9.76%) |
|  |  | BID-2.5 | 38 (46.34%) |
|  |  | BID-5 | 36 (43.90%) |
|  |  | Missing | 0 (0.00 %) |
|  |  |  |  |
|  | **Treatment duration [days]** | N | 5 |
|  |  | Mean (SD) | 228.00 (80.79) |
|  |  | Median [Q1 - Q3] | 263.00 [205.00,280.00] |
|  |  | Min-Max | 97.00 - 295.00 |
|  |  | Missing | 77 (93.90 %) |
|  |  |  |  |
|  | **Time from AF diagnosis to treatment start [days]** | N | 82 |
|  |  | Mean (SD) | 9.54 (17.64) |
|  |  | Median [Q1 - Q3] | 3.00 [1.25,6.75] |
|  |  | Min-Max | 1.00 - 82.00 |
|  |  | Missing | 0 (0.00 %) |
|  |  |  |  |
| **Rivaroxaban** |  | N | 48 (33.10 %) |
|  |  |  |  |
|  | **Frequency** | N | 48 |
|  |  | QD | 48 (100.00%) |
|  |  | BID | 0 (0.00%) |
|  |  | TID | 0 (0.00%) |
|  |  | QID | 0 (0.00%) |
|  |  | PRN | 0 (0.00%) |
|  |  | Other | 0 (0.00%) |
|  |  | Missing | 0 (0.00%) |
|  |  |  |  |
|  | **Total daily dose** | N | 48 |
|  |  | Mean (SD) | 18.96 (2.05) |
|  |  | Median [Q1 - Q3] | 20.00 [20.00,20.00] |
|  |  | Min-Max | 15.00 - 20.00 |
|  |  | Missing | 0 (0.00 %) |
|  |  |  |  |
|  | **Dosing schedule (frequency-mg)** | N | 48 |
|  |  | QD-15 | 10 (20.83%) |
|  |  | QD-20 | 38 (79.17%) |
|  |  | Missing | 0 (0.00 %) |
|  |  |  |  |
|  | **Treatment duration [days]** | N | 7 |
|  |  | Mean (SD) | 189.14 (141.19) |
|  |  | Median [Q1 - Q3] | 122.00 [87.50,298.50] |
|  |  | Min-Max | 31.00 - 399.00 |
|  |  | Missing | 41 (85.42 %) |
|  |  |  |  |
|  | **Time from AF diagnosis to treatment start [days]** | N | 48 |
|  |  | Mean (SD) | 9.77 (18.96) |
|  |  | Median [Q1 - Q3] | 3.00 [1.75,6.50] |
|  |  | Min-Max | 1.00 - 85.00 |
|  |  | Missing | 0 (0.00 %) |
|  |  |  |  |
| **Dabigatran** |  | N | 15 (10.34 %) |
|  |  |  |  |
|  | **Frequency** | N | 15 |
|  |  | QD | 0 (0.00%) |
|  |  | BID | 15 (100.00%) |
|  |  | TID | 0 (0.00%) |
|  |  | QID | 0 (0.00%) |
|  |  | PRN | 0 (0.00%) |
|  |  | Other | 0 (0.00%) |
|  |  | Missing | 0 (0.00%) |
|  |  |  |  |
|  | **Total daily dose** | N | 15 |
|  |  | Mean (SD) | 246.67 (39.04) |
|  |  | Median [Q1 - Q3] | 220.00 [220.00,300.00] |
|  |  | Min-Max | 220.00 - 300.00 |
|  |  | Missing | 0 (0.00 %) |
|  |  |  |  |
|  | **Dosing schedule (frequency-mg)** | N | 15 |
|  |  | BID-55 | 1 (6.67%) |
|  |  | BID-75 | 1 (6.67%) |
|  |  | BID-110 | 9 (60.00%) |
|  |  | BID-150 | 4 (26.67%) |
|  |  | Missing | 0 (0.00%) |
|  |  |  |  |
|  | **Treatment duration [days]** | N | 1 |
|  |  | Mean (SD) | 182.00 (0.00) |
|  |  | Median [Q1 - Q3] | 182.00 [182.00,182.00] |
|  |  | Min-Max | 182.00 - 182.00 |
|  |  | Missing | 14 (93.33 %) |
|  |  |  |  |
|  | **Time from AF diagnosis to treatment start [days]** | N | 15 |
|  |  | Mean (SD) | 5.80 (10.18) |
|  |  | Median [Q1 - Q3] | 3.00 [2.00,5.00] |
|  |  | Min-Max | 1.00 - 42.00 |
|  |  | Missing | 0 (0.00 %) |
|  |  |  |  |
| **Oral Anticoagulant: VKA** |  | N | 17 (6.54 %) |
| **Warfarin** |  | N | 17 (100.00 %) |
|  |  |  |  |
|  | **Frequency** | N | 17 |
|  |  | QD | 17 (100.00%) |
|  |  | BID | 0 (0.00%) |
|  |  | TID | 0 (0.00%) |
|  |  | QID | 0 (0.00%) |
|  |  | PRN | 0 (0.00%) |
|  |  | Other | 0 (0.00%) |
|  |  | Missing | 0 (0.00%) |
|  |  |  |  |
|  | **Total daily dose** | N | 17 |
|  |  | Mean (SD) | 3.85 (2.25) |
|  |  | Median [Q1 - Q3] | 3.00 [2.00,5.00] |
|  |  | Min-Max | 1.00 - 10.00 |
|  |  | Missing | 0 (0.00 %) |
|  |  |  |  |
|  | **Dosing schedule (frequency-mg)** | N | 17 |
|  |  | QD-1 | 2 (11.76%) |
|  |  | QD-2 | 1 (5.88%) |
|  |  | QD-2.5 | 3 (17.65%) |
|  |  | QD-3 | 6 (35.29%) |
|  |  | QD-5 | 1 (5.88%) |
|  |  | QD-6 | 1 (5.88%) |
|  |  | QD-10 | 3 (17.65%) |
|  |  | Missing | 0 (0.00 %) |
|  |  |  |  |
|  | **Treatment duration [days]** | N | 3 |
|  |  | Mean (SD) | 59.67 (98.17) |
|  |  | Median [Q1 - Q3] | 5.00 [3.00,89.00] |
|  |  | Min-Max | 1.00 - 173.00 |
|  |  | Missing | 14 (82.35 %) |
|  |  |  |  |
|  | **Time from AF diagnosis to treatment start [days]** | N | 17 |
|  |  | Mean (SD) | 8.82 (12.32) |
|  |  | Median [Q1 - Q3] | 3.00 [1.00,8.00] |
|  |  | Min-Max | 1.00 - 36.00 |
|  |  | Missing | 0 (0.00 %) |
|  |  |  |  |
| **Antiplatelet therapy** |  | N | 84 (32.31 %) |
| **Clopidogrel** |  | N | 39 (46.43 %) |
|  |  |  |  |
|  | **Frequency** | N | 38 |
|  |  | QD | 38 (100.00%) |
|  |  | BID | 0 (0.00%) |
|  |  | TID | 0 (0.00%) |
|  |  | QID | 0 (0.00%) |
|  |  | PRN | 0 (0.00%) |
|  |  | Other | 0 (0.00%) |
|  |  | Missing | 1 (2.56%) |
|  |  |  |  |
|  | **Total daily dose** | N | 38 |
|  |  | Mean (SD) | 75.00 (0.00) |
|  |  | Median [Q1 - Q3] | 75.00 [75.00,75.00] |
|  |  | Min-Max | 75.00 - 75.00 |
|  |  | Missing | 1 (2.56 %) |
|  |  |  |  |
|  | **Dosing schedule (frequency-mg)** | N | 38 |
|  |  | QD-75 | 38 (100.0%) |
|  |  | Missing | 0 (0.00%) |
|  |  |  |  |
|  | **Treatment duration [days]** | N | 7 |
|  |  | Mean (SD) | 452.14 (709.48) |
|  |  | Median [Q1 - Q3] | 254.00 [64.00,376.50] |
|  |  | Min-Max | 7.00 - 2023.00 |
|  |  | Missing | 32 (82.05 %) |
|  |  |  |  |
|  | **Time from AF diagnosis to treatment start [days]** | N | 39 |
|  |  | Mean (SD) | -130.41 (413.91) |
|  |  | Median [Q1 - Q3] | 1.00 [1.00,2.00] |
|  |  | Min-Max | -1845.00 - 6.00 |
|  |  | Missing | 0 (0.00 %) |
|  |  |  |  |
| **Aspirin** |  | N | 42 (50.00 %) |
|  |  |  |  |
|  | **Frequency** | N | 41 |
|  |  | QD | 41 (100.00%) |
|  |  | BID | 0 (0.00%) |
|  |  | TID | 0 (0.00%) |
|  |  | QID | 0 (0.00%) |
|  |  | PRN | 0 (0.00%) |
|  |  | Other | 0 (0.00%) |
|  |  | Missing | 1 (2.38%) |
|  |  |  |  |
|  | **Total daily dose** | N | 41 |
|  |  | Mean (SD) | 89.63 (13.17) |
|  |  | Median [Q1 - Q3] | 100.00 [75.00,100.00] |
|  |  | Min-Max | 51.00 - 100.00 |
|  |  | Missing | 1 (2.38 %) |
|  |  |  |  |
|  | **Dosing schedule (frequency-mg)** | N | 41 |
|  |  | QD-51 | 1 (2.44%) |
|  |  | QD-75 | 12 (29.27%) |
|  |  | QD-81 | 4 (9.76%) |
|  |  | QD-100 | 24 (58.54%) |
|  |  | Missing | 0 (0.00%) |
|  |  |  |  |
|  | **Treatment duration [days]** | N | 4 |
|  |  | Mean (SD) | 31.00 (38.11) |
|  |  | Median [Q1 - Q3] | 14.00 [11.75,33.25] |
|  |  | Min-Max | 8.00 - 88.00 |
|  |  | Missing | 38 (90.48 %) |
|  |  |  |  |
|  | **Time from AF diagnosis to treatment start [days]** | N | 42 |
|  |  | Mean (SD) | -229.29 (680.91) |
|  |  | Median [Q1 - Q3] | 1.00 [1.00,1.00] |
|  |  | Min-Max | -3671.00 - 8.00 |
|  |  | Missing | 0 (0.00 %) |
|  |  |  |  |
| **Ticagrelor** |  | N | 2 (2.38 %) |
|  |  |  |  |
|  | **Frequency** | N | 2 |
|  |  | QD | 0 (0.00%) |
|  |  | BID | 2 (100.00%) |
|  |  | TID | 0 (0.00%) |
|  |  | QID | 0 (0.00%) |
|  |  | PRN | 0 (0.00%) |
|  |  | Other | 0 (0.00%) |
|  |  | Missing | 0 (0.00%) |
|  |  |  |  |
|  | **Total daily dose** | N | 2 |
|  |  | Mean (SD) | 180.00 (0.00) |
|  |  | Median [Q1 - Q3] | 180.00 [180.00,180.00] |
|  |  | Min-Max | 180.00 - 180.00 |
|  |  | Missing | 0 (0.00 %) |
|  |  |  |  |
|  | **Dosing schedule (frequency-mg)** | N | 2 |
|  |  | BID-90 | 2 (100.0%) |
|  |  | Missing | 0 (0.00%) |
|  |  |  |  |
|  |  |  |  |
|  | **Treatment duration [days]** | N | 1 |
|  |  | Mean (SD) | 182.00 (0.00) |
|  |  | Median [Q1 - Q3] | 182.00 [182.00,182.00] |
|  |  | Min-Max | 182.00 - 182.00 |
|  |  | Missing | 1 (50.00 %) |
|  |  |  |  |
|  | **Time from AF diagnosis to treatment start [days]** | N | 2 |
|  |  | Mean (SD) | 3.50 (0.71) |
|  |  | Median [Q1 - Q3] | 3.50 [3.25,3.75] |
|  |  | Min-Max | 3.00 - 4.00 |
|  |  | Missing | 0 (0.00 %) |
|  |  |  |  |
| **Dipyridamole** |  | N | 1 (1.19 %) |
|  |  |  |  |
|  | **Frequency** | N | 1 |
|  |  | QD | 1 (100.00%) |
|  |  | BID | 0 (0.00%) |
|  |  | TID | 0 (0.00%) |
|  |  | QID | 0 (0.00%) |
|  |  | PRN | 0 (0.00%) |
|  |  | Other | 0 (0.00%) |
|  |  | Missing | 0 (0.00%) |
|  |  |  |  |
|  | **Total daily dose** | N | 1 |
|  |  | Mean (SD) | 75.00 (0.00) |
|  |  | Median [Q1 - Q3] | 75.00 [75.00,75.00] |
|  |  | Min-Max | 75.00 - 75.00 |
|  |  | Missing | 0 (0.00 %) |
|  |  |  |  |
|  | **Dosing schedule (frequency-mg)** | N | 1 |
|  |  | QD-75 | 1 (100.00%) |
|  |  | Missing | 0 (0.00 %) |
|  |  |  |  |
|  |  |  |  |
|  | **Treatment duration [days]** | N | 0 |
|  |  | Mean (SD) |  |
|  |  | Median [Q1 - Q3] |  |
|  |  | Min-Max |  |
|  |  | Missing | 1 (100.00 %) |
|  |  |  |  |
|  | **Time from AF diagnosis to treatment start [days]** | N | 1 |
|  |  | Mean (SD) | -1540.00 (0.00) |
|  |  | Median [Q1 - Q3] | -1540.00 [-1540.00,-1540.00] |
|  |  | Min-Max | -1540.00 - -1540.00 |
|  |  | Missing | 0 (0.00 %) |
|  |  |  |  |
| **Other antithrombotic therapy** |  | N | 13 (5.00 %) |
|  |  |  |  |
|  | **Frequency** | N | 13 |
|  |  | QD | 5 (38.46%) |
|  |  | BID | 8 (61.54%) |
|  |  | TID | 0 (0.00%) |
|  |  | QID | 0 (0.00%) |
|  |  | PRN | 0 (0.00%) |
|  |  | Other | 0 (0.00%) |
|  |  | Missing | 0 (0.00%) |
|  |  |  |  |
|  |  |  |  |
|  | **Total daily dose [mg]** | N | 13 |
|  |  | Mean (SD) | 66.69 (16.19) |
|  |  | Median [Q1 - Q3] | 60.00 [60.00,80.00] |
|  |  | Min-Max | 40.00 - 90.00 |
|  |  | Missing | 0 (0.00 %) |
|  |  |  |  |
|  | **Dosing schedule (frequency-mg)** | N | 13 |
|  |  | QD-40 | 2 (15.38%) |
|  |  | QD-60 | 2 (15.38%) |
|  |  | QD-80 | 1 (7.69%) |
|  |  | BID-30 | 3 (23.08%) |
|  |  | BID-35 | 1 (7.69%) |
|  |  | BID-40 | 3 (23.08%) |
|  |  | BID-45 | 1 (7.69%) |
|  |  | Missing | 0 (0.00%) |
|  |  |  |  |
|  | **Total daily dose [units]** | N | 0 |
|  |  | Mean (SD) |  |
|  |  | Median [Q1 - Q3] |  |
|  |  | Min-Max |  |
|  |  | Missing |  |
|  |  |  |  |
|  | **Treatment duration [days]** | N | 10 |
|  |  | Mean (SD) | 5.70 (5.58) |
|  |  | Median [Q1 - Q3] | 4.00 [2.25,5.50] |
|  |  | Min-Max | 2.00 - 20.00 |
|  |  | Missing | 3 (23.08 %) |
|  |  |  |  |
|  | **Time from AF diagnosis to treatment start [days]** | N | 13 |
|  |  | Mean (SD) | 1.85 (1.77) |
|  |  | Median [Q1 - Q3] | 1.00 [1.00,4.00] |
|  |  | Min-Max | -1.00 - 5.00 |
|  |  | Missing | 0 (0.00 %) |
|  |  |  |  |

*Treatment group is based on recorded drug name. In the "Other concomitant medications log" 310 entries were found to refer to antithrombotic medications without being recorded in the "antithrombotic medications log” and are thus used in this count. The total number of antithrombotic drugs not recorded in the "antithrombotic drug form" is unknown.*
